# Supplementary material for: A pause in the weakening of the Atlantic meridional overturning circulation since the early 2010s
Source: Nat Commun. 2024 Dec 6;15:10642. doi: 10.1038/s41467-024-54903-w (PMC11624277; doi:10.1038/s41467-024-54903-w)
Supplement: Supplementary file 1 — Supplementary Information [file 41467_2024_54903_MOESM1_ESM.pdf]

nature communications

Supplementary Information for
A pause in the weakening of the Atlantic meridional overturning circulation since the early 2010s

Sang-Ki Lee[1], Dongmin Kim[2,1], Fabian A. Gomez[3,1], Hosmay Lopez[1], Denis L. Volkov[2,1], Shenfu Dong[1], Rick Lumpkin[1], and Stephen Yeager[4]

[1]NOAA Atlantic Oceanographic and Meteorological Laboratory, Miami, FL, USA
[2]Cooperative Institute for Marine and Atmospheric Studies, University of Miami, Miami, FL, USA
[3]Northern Gulf Institute, Mississippi State University, Mississippi State, MS, USA
[4]National Center for Atmospheric Research, Boulder, CO, USA

Supplementary Note 1. Sensitivity in the interdecadal variation of the AMOC to model resolution in OMIP2 models

The attribution of the interdecadal AMOC variation presented in Figures 1 and 2 is not exclusive and to some extent model dependent. This issue of model dependency is addressed to some degree in this study by analyzing multiple OMIP2 and CMIP6 models along with two large ensemble simulations (CESM2 and SPEAR). To further explore this point, we carried out supplementary analysis using additional OMIP2 and CMIP6 models.

The multi model-mean OMIP2 indicates an increase in the total AMOC from 1958-64 to 1995-2004 followed by a decrease after the 1995-2004 peak (Figure 1). However, as shown in Supplementary Figure 1, large inter-model spread exists in the OMIP2 models. In particular, out of the total 10 OMIP2 models, six have the AMOC peak in 1985-94, three have the peak in 1995-2004, and one has the peak in 2005-14.

Previous studies reported that low resolution OMIP2 models tend to produce weaker than observed AMOC intensity while the AMOC intensity in high resolution OMIP2 models agrees better with observations[1,2]. Thus, we next explore if there are any systematic differences in the interdecadal AMOC swing between high and low resolution OMIP2 models. Specifically, we compare four sets of high (~10 *km*) and low (~100 *km*) resolution OMIP2 models discussed in ref 1 (Supplementary Table 5). As shown in Supplementary Figure 2, the high resolution OMIP2 models tend to show higher amplitude interdecadal swing compared to the low resolution OMIP2 models. However, both the high and low resolution versions of the four OMIP2 models have the same tendency to display the AMOC peak in 1985-94 rather than 1995-2004, although inter-model spread is quite large (Supplementary Figure 3).

In summary, based on the results shown in Supplementary Figures 1-3, it appears that high-resolution OMIP2 models tend to show higher amplitude interdecadal AMOC swing. However, the timing of the AMOC peak (1985-94 or 1995-2004) in OMIP2 models is largely model dependent, and its dependency to model resolution is unclear.

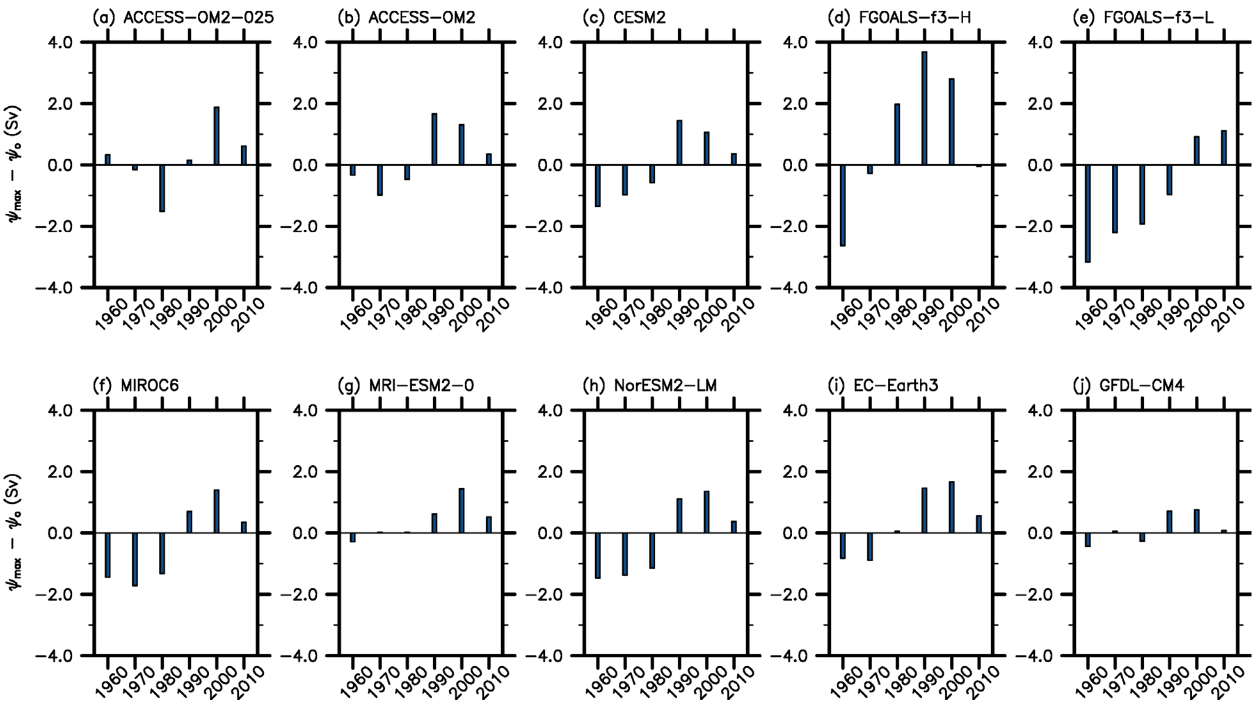

Supplementary Figure 1. Decade-averaged time series of the AMOC anomalies at 26.5°N from 10 OMIP2 models. Decade-averaged time series of the AMOC anomalies at 26.5°N from the decade centered in 1960 (i.e., 1958-64) to the decade centered in 2010 (i.e., 2005-14), derived from ten OMIP2 models used in this study.

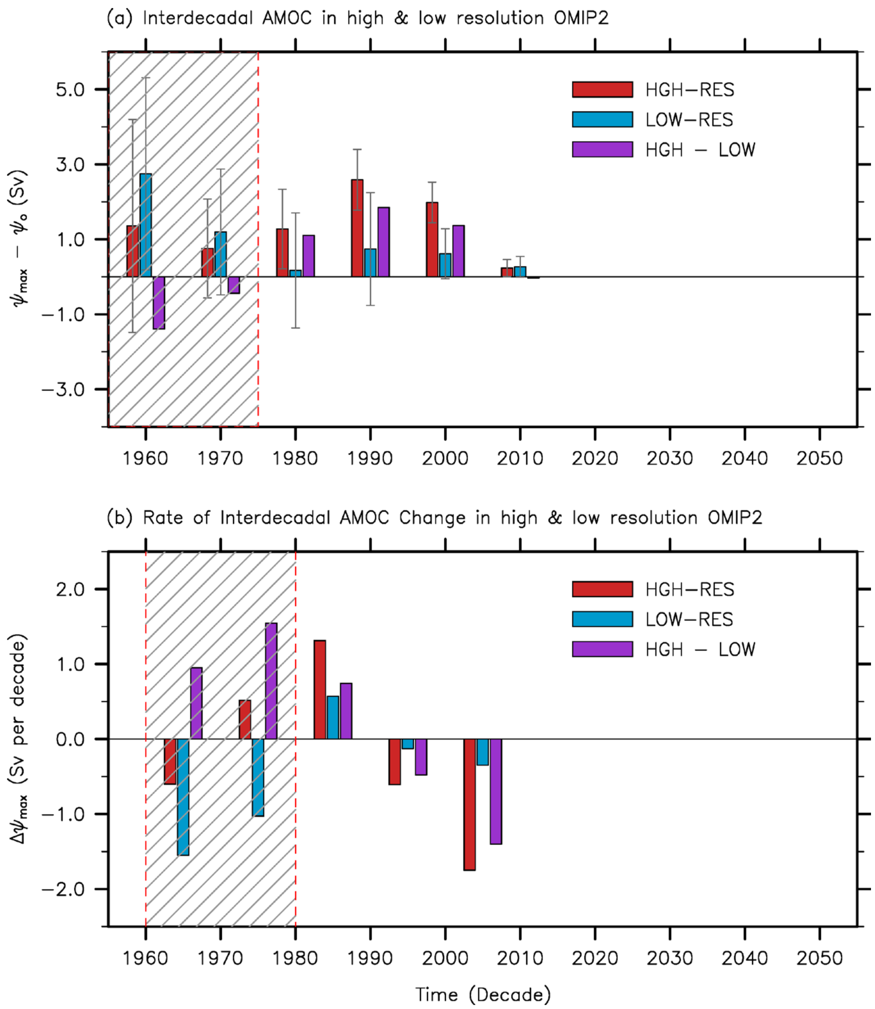

Supplementary Figure 2. Decade-averaged time series of the AMOC anomalies at 26.5°N in high and low resolution OMIP2 models. (a) Decade-averaged time series of the AMOC anomalies at 26.5°N from the decade centered in 1960 (i.e., 1958-64) to the decade centered in 2010 (2005-14), derived from four sets of high and low resolution OMIP2 models discussed in ref 1. (b) Same as (a) except that the rate of interdecadal AMOC change is shown. The error bars in (a) indicate standard deviation from the ensemble-mean. Note that OMIP2 model runs are typically carried out for 366 years by repeating six cycles of the 61-year (1958-2018). However, for the high and low resolution OMIP2 simulations used in ref 1, no spin-up run was carried out. Hence, the AMOC time series during the first 17 years (i.e., 1958-74) are stippled because they are contaminated by spin-up issues[1], and thus should be disregarded.

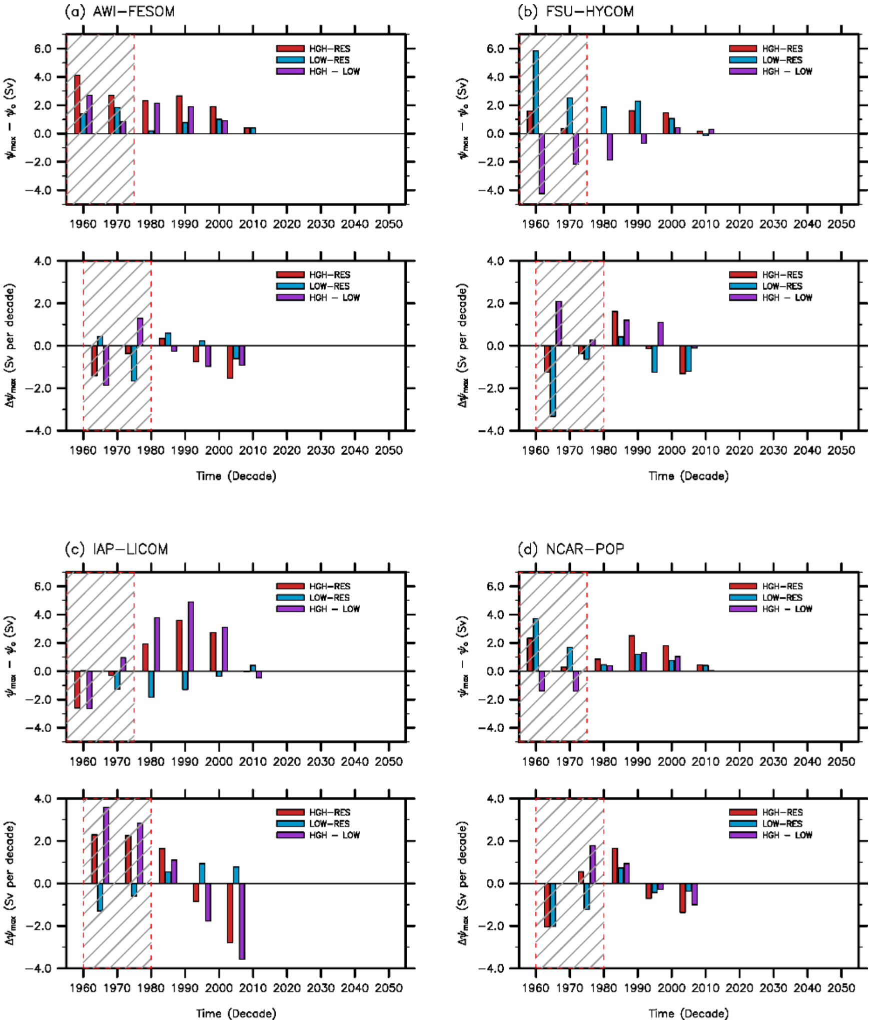

Supplementary Figure 3. Decade-averaged time series of the AMOC anomalies at 26.5°N in four sets of high and low resolution OMIP2 models. Decade-averaged time series of the AMOC anomalies at 26.5°N from the decade centered in 1960 (i.e., 1958-64) to the decade centered in 2010 (i.e., 2005-14), derived from four sets of high and low resolution OMIP2 models: (a) AWI-FESOM, (b) FSU-HYCOM, (c) IAP-LICOM, and (d) NCAR-POP. The rate of interdecadal AMOC change for each OMIP2 model is also shown in the lower penal. The AMOC time series during the first 17 years (i.e., 1958-74) are stippled because they are contaminated by spin-up issues[1], and thus should be disregarded.

Supplementary Note 2. Sensitivity in attributing the interdecadal AMOC variation to model resolution in CMIP6 models

We next explore the potential effects of horizontal resolution on the externally forced interdecadal AMOC signal in CMIP6 models. To do so, it is important to use CMIP6 models with multiple ensemble members available in both high (or medium) and low resolutions. Thus, here we use the HadGEM3-GC31 simulations under the historical and SSP-585 scenarios[3], available in both low (~100 *km*) and medium (~25 *km*) resolutions with 4 ensemble members for both resolutions (Supplementary Table 6).

As shown in Supplementary Figure 4, the externally forced interdecadal AMOC signal in the low resolution runs (HadGEM3-GC31-LL) is much weaker than that in the medium resolution runs (HadGEM3-GC31-MM). The weaker externally forced AMOC signal in HadGEM3-GC31-LL appears to be more consistent with that of the multi model-mean CMIP6 (Figures 2a & 2b), whereas the stronger externally forced AMOC signal in HadGEM3-GC31-MM appears to be more consistent with that of the ensemble-mean CESM2 (Figures 1a & 1b) and the ensemble-mean SPEAR (Figures 2c & 2d). The externally forced AMOC in HadGEM3-GC31-LL displays its maximum in 1985-94 and a slightly weaker amplitude in 1975-84. In HadGEM3-GC31-MM, the externally forced AMOC displays its maximum in 1975-84 and a slightly weaker amplitude in 1985-94. This result is overall consistent with the externally forced AMOC signals in CESM2 (Figure 1a; the maximum in 1975-84 and 1985-94), CMIP6 (Figure 2a; the maximum in 1975-84) and SPEAR (Figure 2c; the maximum in 1975-84).

In summary, the additional analysis with the low and medium resolution versions of HadGEM3-GC31 indicates that the overall interdecadal evolutions of the externally forced and residual AMOC components in the low and medium resolution versions of HadGEM3-GC31 (Supplementary Figure 4) are largely consistent with those in CESM2, CMIP6 and SPEAR (Figures 1 and 2). However, the externally forced interdecadal AMOC signal is much weaker in the low resolution runs than that in the medium resolution runs. Hence, the interdecadal swing of the residual AMOC component is also weaker in the low resolution runs than that in the medium resolution runs (Supplementary Figure 5).

Therefore, our analysis with additional OMIP2 and CMIP6 models affirms that the attribution of the interdecadal AMOC variation is not exclusive and to some degree model dependent. Specifically, high (~10 *km*) resolution OMIP2 models and medium resolution (~25 *km*) CMIP6 models tend to show higher amplitude interdecadal swing of the total and externally forced AMOC components, respectively compared to low resolution (~100 *km*) OMIP2 and CMIP6 models, although inter-model spread is quite large.

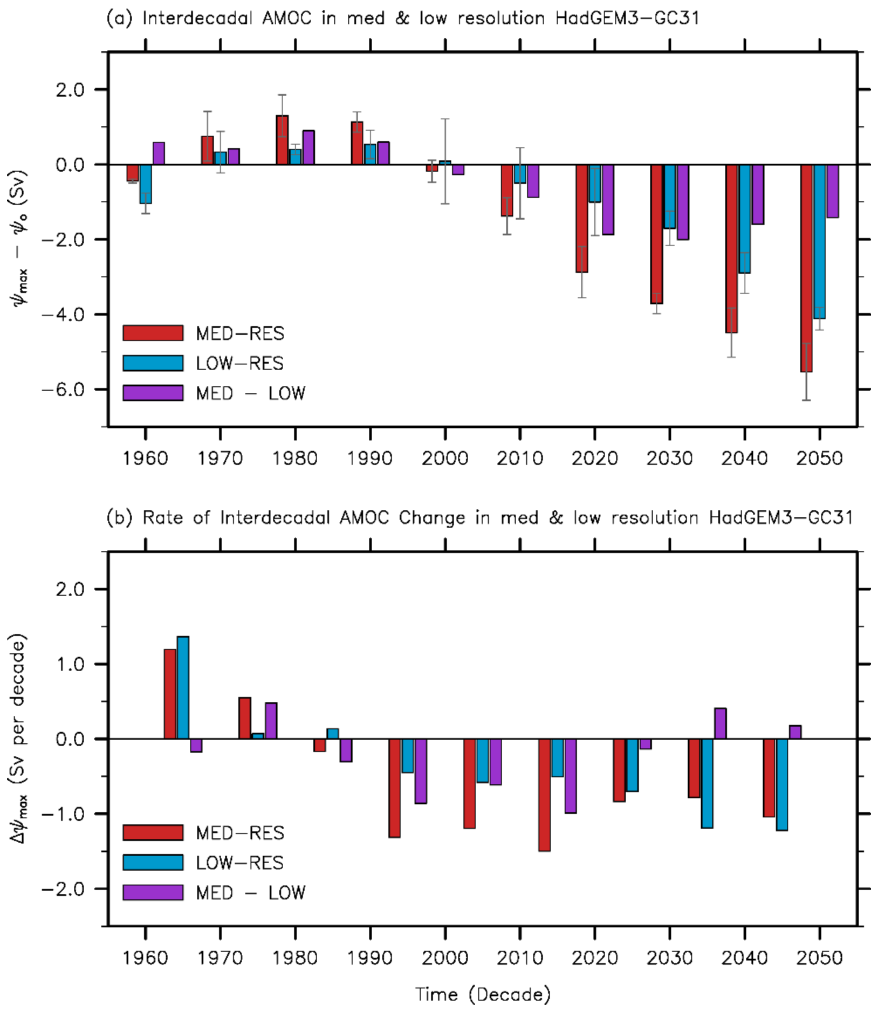

Supplementary Figure 4. Interdecadal time series of the externally forced AMOC and its rate of change at 26.5°N in low and medium resolution HadGEM3-GC31. (a) Interdecadal time series of the externally forced AMOC and (b) its rate of change at 26.5°N derived from HadGEM3-GC31-MM (red), HadGEM3-GC31-LL (sky blue) and the difference between the two (purple). The error bars in (a) indicate standard deviation from the ensemble-mean.

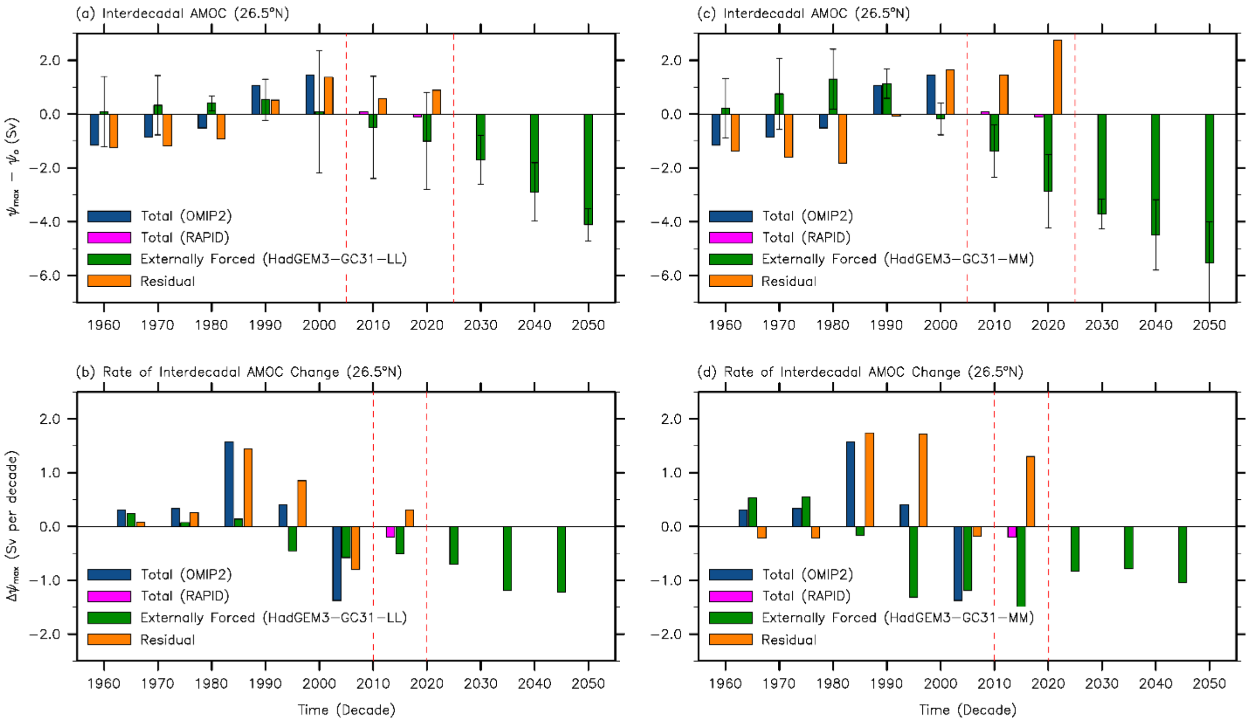

Supplementary Figure 5. Interdecadal time series of the AMOC and its rate of change at 26.5°N based on low and medium resolution versions of HadGEM3-GC31. (a,b) Interdecadal time series of the AMOC and (b,d) its rate of change at 26.5°N based on (a,b) HadGEM3-GC31-LL and (c,d) HadGEM3-GC31-MM. The error bars in (a,c) indicate standard deviation from the ensemble-mean.

Supplementary Note 3. Further discussion on the December-May NAO index

In this study, the difference in sea level pressure anomalies between the Azores high (36° – 40°N and 28W°28°W – 20°W) and Icelandic low regions (63° – 70°N and 25° – 16°W) derived from ERA5 is used to compute December-May NAO time series. To examine the sensitivity of the NAO index to the choice of datasets and computation methods, we derive several alternative sets of December-May NAO time series using sea level pressure anomalies from the National Center for Environmental Prediction - National Center for Atmospheric Research Reanalysis -1 (NCEP1)[4], and the Japanese 55-year Reanalysis (JRA55)[5], and applying an Empirical Orthogonal Function (EOF) analysis[6] to sea level pressure anomalies in the North Atlantic (0° - 80°N and 90°W - 0°). In both NCEP1 and JRA55, the NAO is represented as the first EOF mode. In ERA5, the NAO is represented as the second EOF mode.

As summarized in Supplementary Figure 6, the spatiotemporal NAO patterns derived from NCEP1 and JRA55 and those computed using the EOF-based definition of the NAO index are largely consistent with those derived from ERA5 and the area-averaged definition of the NAO index. Some noticeable differences are seen only in the EOF-based ERA5 NAO time series prior to the late 1960s and also around the mid-to-late 1990s to a lesser degree.

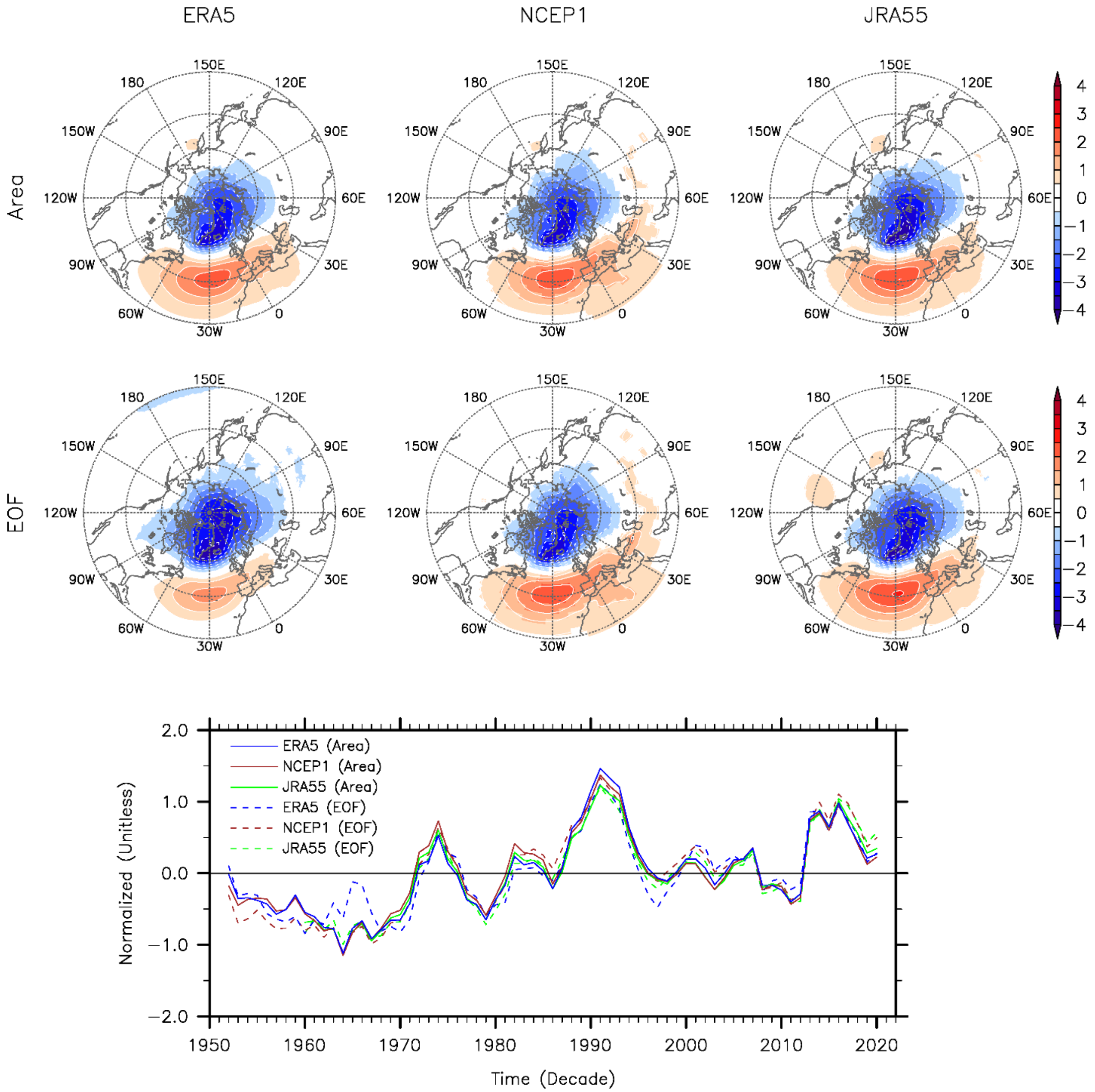

Supplementary Figure 6. Spatiotemporal patterns of the area-averaged and EOF-based December-May NAO. (top panels) Spatial patterns of the area-averaged December-May NAO derived from (left) ERA5, (middle) NCEP1, and (right) JRA55. (middle panels) Spatial patterns of the EOF-based December-May NAO derived from (left) ERA5, (middle) NCEP1, and (right) JRA55. (bottom panel) 5-year running-mean time series of the area-averaged (solid lines) and EOF-based (dashed lines) December-May NAO derived from ERA5 (blue), NCEP1 (red), and JRA55 (green). The units for sea level pressure anomalies in the upper and middle panels are *hPa* per one standard deviation of the NAO.

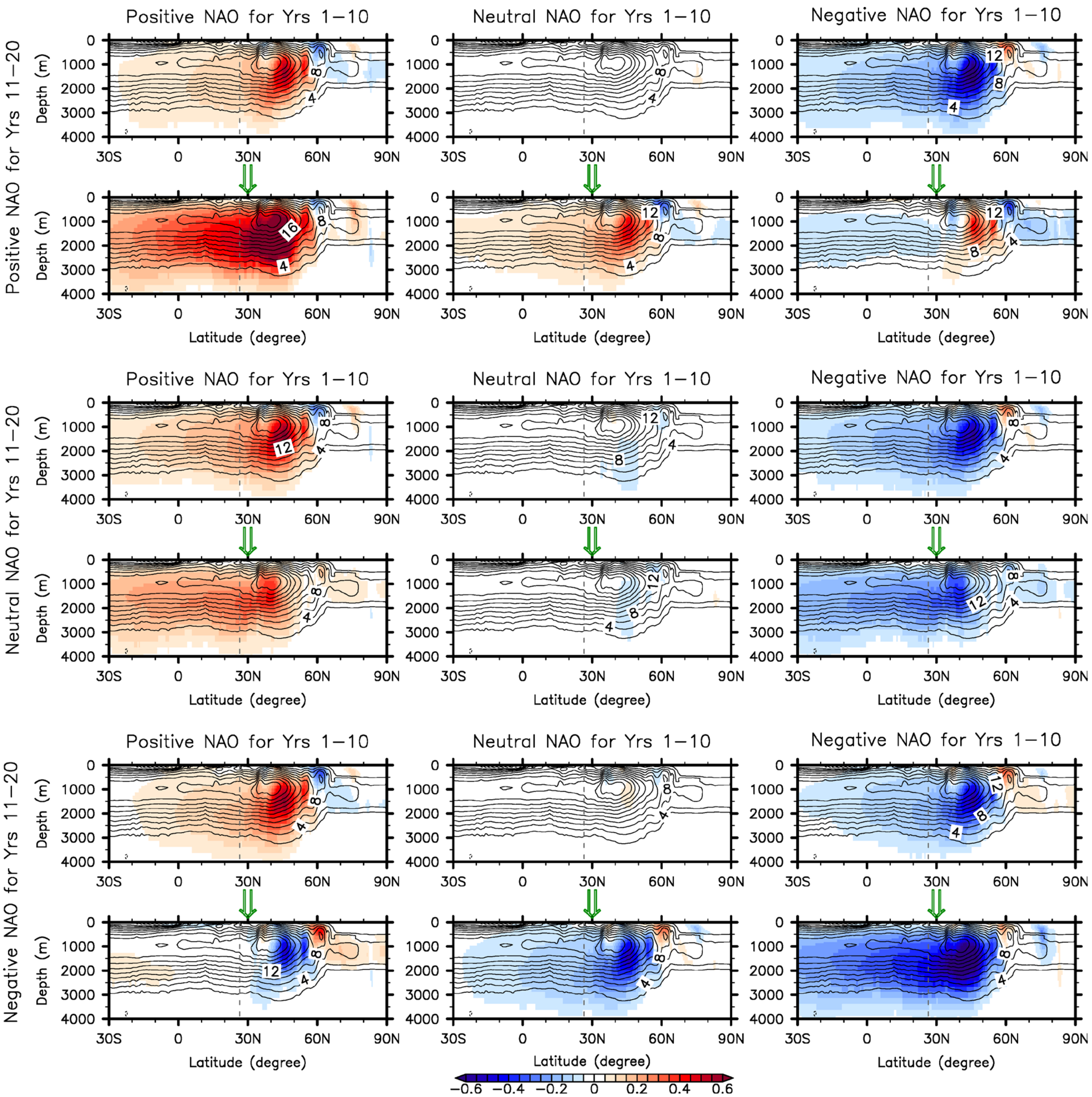

Supplementary Figure 7. Residual AMOC component response to interdecadal NAO variability. The composite maps of residual AMOC component response to nine different possible cases of interdecadal NAO variability derived from CESM2. The units are *Sv* (10^6 $m^3 sec^{-1}$).

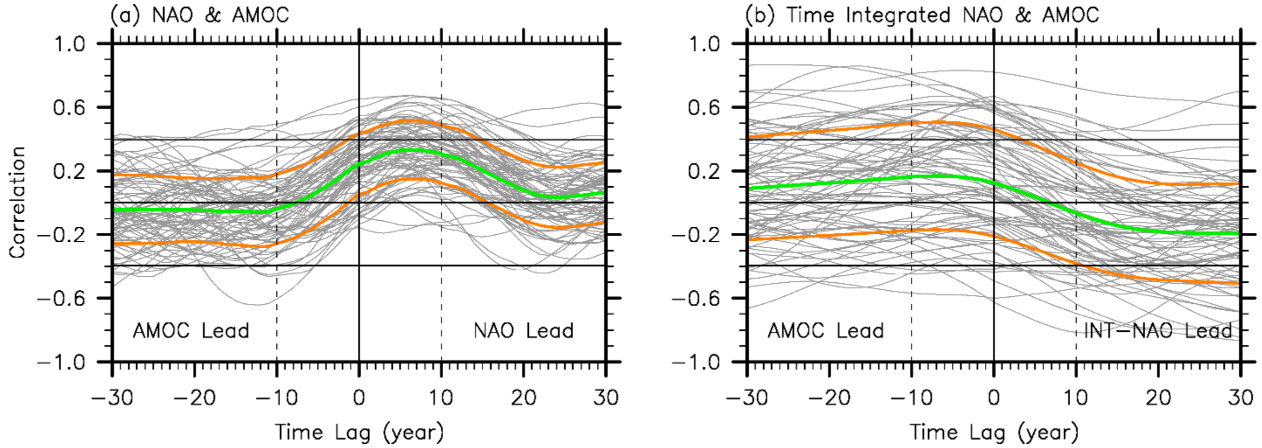

Supplementary Figure 8. Lead-lag correlations of the NAO, the time-integrated NAO and the residual AMOC component at 26.5°N. Lead-lag correlations (a) between the NAO and the residual AMOC component at 26.5°N, and (b) between the time-integrated NAO and the residual AMOC component at 26.5°N derived from CESM2. The green lines in (a,b) indicate the ensemble-averaged correlation values. The orange lines in (a,b) indicate the ensemble spread (i.e., standard deviation). Thick horizontal lines in (a,b) indicate the 95% confidence intervals (based on a Student-t test) and zero correlations. The degree of freedom for the Student-t test is determined by the number of decades since the NAO, time-integrated NAO and AMOC indices are smoothed by performing a 10-year running-average prior to the correlation analysis to focus on interdecadal time scale. The units for the residual AMOC component are *Sv* (10^6 $m^3 sec^{-1}$).

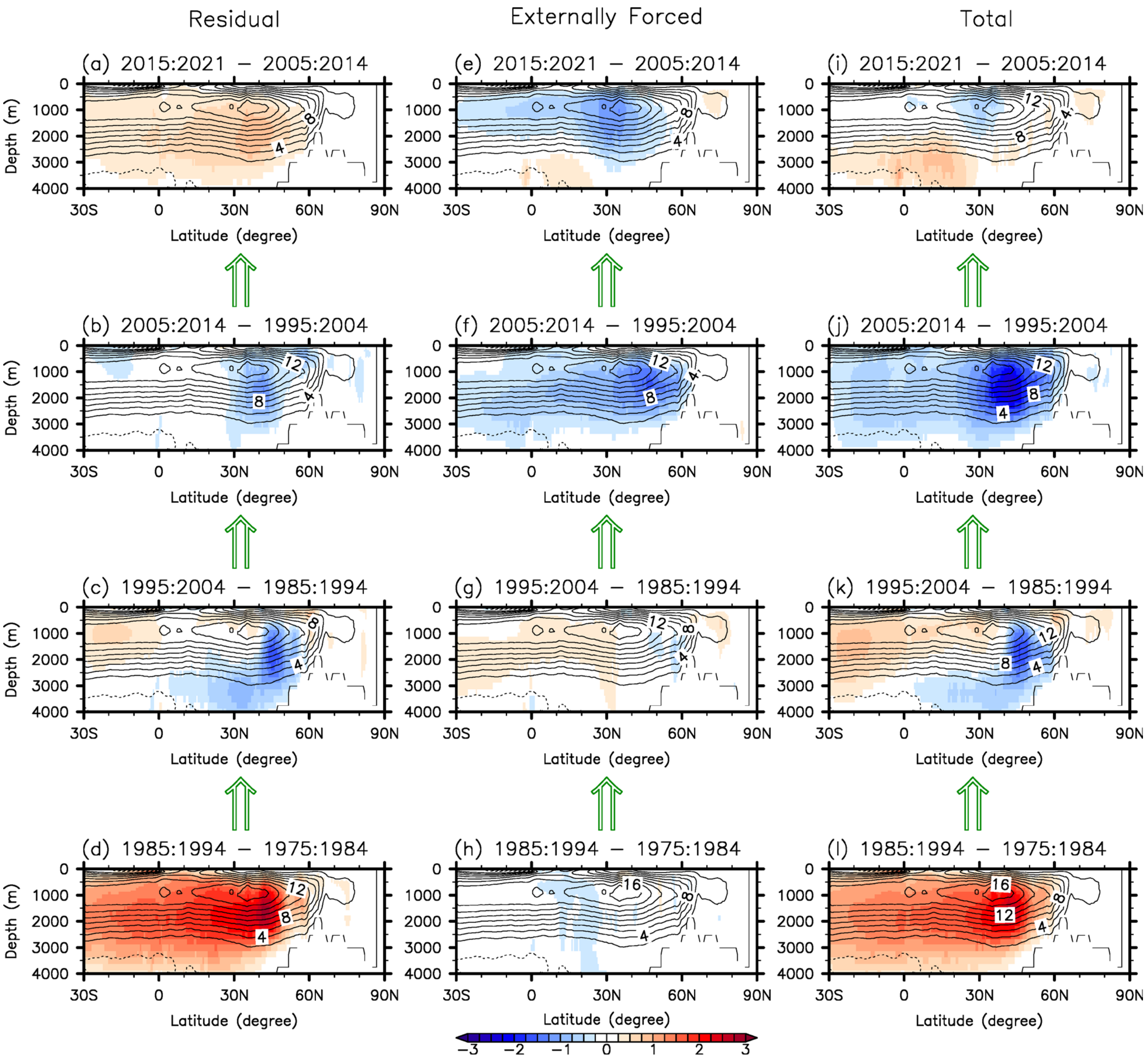

Supplementary Figure 9. Same as Figure 6, but the externally forced AMOC component is derived from CMIP6. The units are *Sv* (10^6 $m^3 sec^{-1}$).

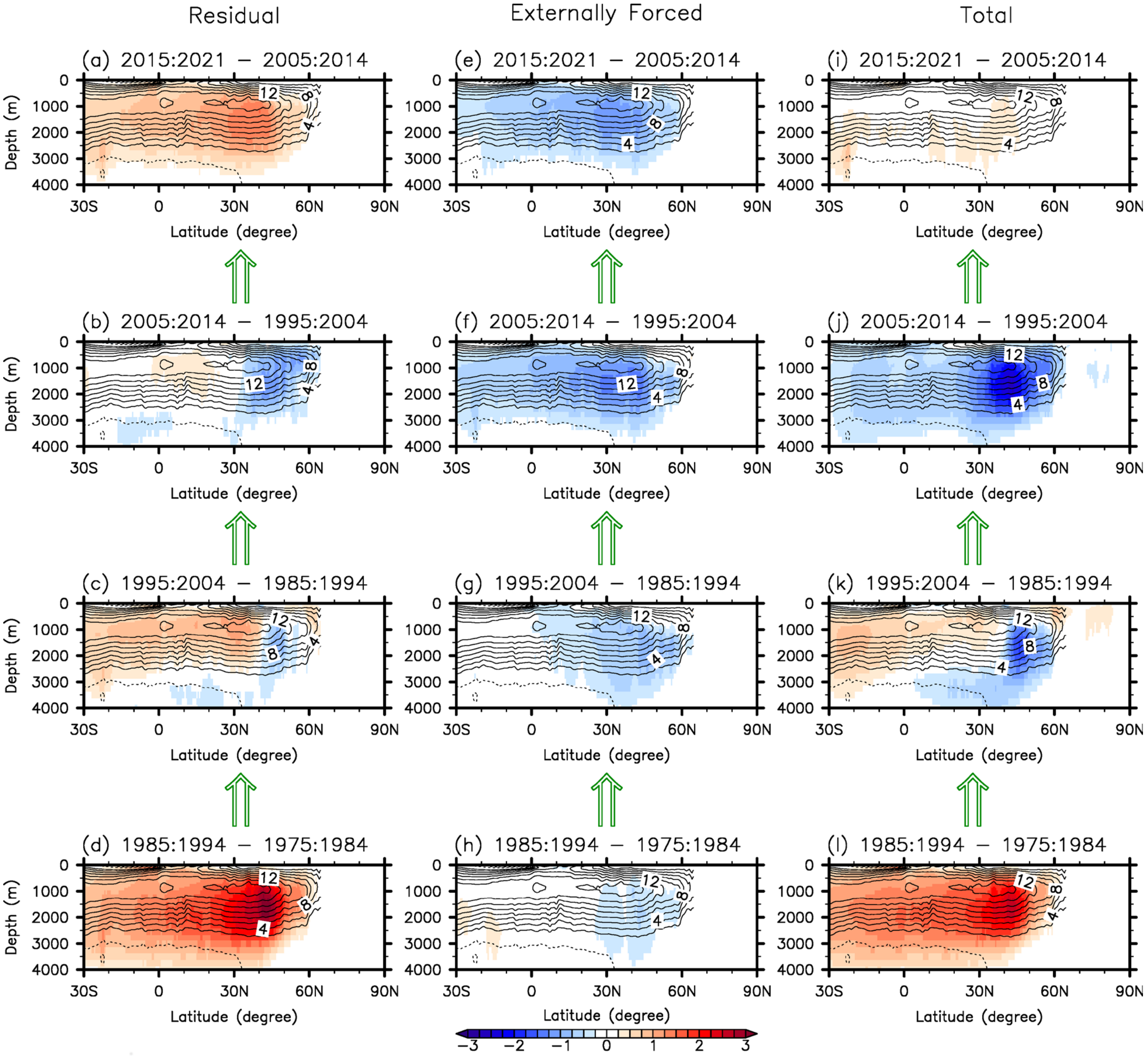

Supplementary Figure 10. Same as Figure 6, but the externally forced AMOC component is derived from SPEAR. The units are *Sv* (10^6 $m^3 sec^{-1}$).

Supplementary Table 1. Summary of OMIP2 models. List of 10 OMIP2 models analyzed in this study and their AMOC values at 26.5°N averaged during 1958-2018. Horizontal and vertical resolutions for each model are also shown. The units are *Sv* (10^6 m^3sec^{-1}) for the AMOC, and *km* for horizontal resolution.

Description / Model	AMOC at 26.5°N (1958-2018)	Horizontal Resolution	Vertical Levels
ACCESS-OM2	14.0	100	50
ACCESS-OM2-025	14.0	25	50
CESM2	14.9	100	60
EC-Earth3	15.1	100	75
FGOALS-f3-H	21.2	10	55
FGOALS-f3-L	15.7	100	30
GFDL-CM4	14.1	25	36
MIROC6	16.0	100	63
MRI-ESM2-0	13.2	100	61
NorESM2-LM	19.5	100	70

Supplementary Table 2. Summary of CESM2, CMIP6 and SPEAR. Summary of the two large ensemble simulations (CESM2 and SPEAR) and CMIP6 models used in this study. The units are Sv (10^6 $m^3 sec^{-1}$) for the AMOC, and *km* for horizontal resolution.

Description / Model	AMOC at 26.5°N (1958-2018)	Number of Ensembles (or Models)	CMIP6 Scenarios	Horizontal Resolution (Atm.)	Horizontal Resolution (Ocean)	Vertical Levels (Ocean)
CESM2	19.6	80	SSP-370	100	100	60
CMIP6	19.1	20	SSP-370	Variable	Variable	Variable
SPEAR	16.7	30	SSP-585	50	100	75

Supplementary Table 3. Summary of CMIP6 models. Summary of 29 CMIP6 models used in this study. An open circle indicates that the corresponding CMIP6 model is used for the specific scenario, while the × symbol indicates that the model is not used. The units are *Sv* (10^6 m^3sec^{-1}) for the AMOC, and *km* for horizontal resolution.

Model \ Description	Pre Industrial (500 years)	Historical (1850-2014)	SSP-370 (2015-2100)	AMOC at 26.5°N (1958-2018)	Horizontal Resolution (Atm.)	Horizontal Resolution (Ocean)	Vertical Levels (Ocean)
ACCESS-CM2	○	○	○	19.5	200	100	50
ACCESS-ESM1-5	○	○	○	19.4	200	100	50
CanESM5	○	○	○	13.6	300	100	45
CanESM5-1	○	○	○	13.0	300	100	45
CAS-ESM2-0	×	○	○	19.5	140	100	30
CESM2	○	×	×	-	125	100	60
CESM2-FV2	○	×	×	-	250	100	60
CESM2-WACCM	○	○	○	19.7	125	100	60
CESM2-WACCM-FV2	○	×	×	-	250	100	60
CMCC-CM2-SR5	○	○	○	17.3	120	100	50
CMCC-ESM2	○	○	○	17.5	120	100	50
E3SM-1-0	○	×	×	-	100	60	60
EC-Earth3-AerChem	○	○	○	19.7	80	100	75
EC-Earth3	○	×	×	-	80	100	75
EC-Earth3-CC	○	×	×	-	80	100	75
FGOALS-f3-L	×	○	○	20.7	100	100	30
FGOALS-g3	×	○	○	27.8	200	100	30
GFDL-ESM4	×	○	○	18.9	100	50	75
HadGEM3-GC31-LL	○	×	×	-	200	100	75
HadGEM3-GC31-MM	○	×	×	-	80	25	75
INM-CM4-8	×	○	○	21.0	200	100	40
INM-CM5-0	○	○	○	19.6	200	50	40
MIROC6	○	○	○	14.4	140	100	63
MPI-ESM-1-2-HAM	○	×	×	-	200	150	40
MPI-ESM1-2-HR	○	○	○	17.2	100	40	40
MPI-ESM1-2-LR	○	○	○	18.7	200	150	40
MRI-ESM2-0	○	○	○	19.2	100	50	61
NorESM2-LM	×	○	○	22.5	200	100	70
NorESM2-MM	×	○	○	22.5	125	100	70

Supplementary Table 4. Expected changes in the AMOC components during 2022-26. Expected changes in the AMOC components (i.e., residual, externally forced, and total) from 2015-21 to 2022-26 derived from CESM2, CMIP6 and SPEAR. The residual AMOC component values for 2022-26 (i.e., 1.7, 1.0, and 1.0 *Sv* for CESM2, CMIP6 and SPEAR, respectively) are derived based on a simple autoregression tool (i.e., Figures 5g-i) applied to the residual AMOC component values for 2015-21 (i.e., 2.2, 1.3, and 1.9 *Sv* for CESM2, CMIP6 and SPEAR, respectively). Error estimates are based on the 95% limits of the regressed residual AMOC component values. The expected total AMOC values for 2022-26 based on the observed value of the AMOC during 2015-21 (i.e., 16.6 *Sv*) are also indicated (values inside parentheses). The units are *Sv* ($10^6\ m^3sec^{-1}$).

Model \ AMOC	Estimated Changes in Residual AMOC Component at 26.5°N	Estimated Changes in Externally forced AMOC at 26.5°N	Estimated Changes in Total AMOC at 26.5°N
CESM2	-0.5 ± 1.1	-0.6	-1.1 ± 1.1 (15.5 ± 1.1)
CMIP6	-0.3 ± 1.1	-0.4	-0.7 ± 1.1 (15.9 ± 1.1)
SPEAR	-0.9 ± 1.3	-0.3	-1.2 ± 1.3 (15.4 ± 1.3)

Supplementary Table 5. Four sets of OMIP2 models with high and low resolution. Four sets of OMIP2 models with high and low resolution and their AMOC values at 26.5°N averaged during 1958-2018. Horizontal and vertical resolutions for each model are also shown. The units are *Sv* (10^6 m^3sec^{-1}) for the AMOC, and *km* for horizontal resolution.

Model \ Description		AMOC at 26.5°N (1958-2018)	Horizontal Resolution	Vertical Levels
AWI-FESOM	Low Res.	13.8	100	46
	High Res.	18.2	10	46
FSU-HYCOM	Low Res.	9.6	72	41
	High Res.	14.6	8	36
IAP-LICOM	Low Res.	13.9	100	30
	High Res.	20.7	10	55
NCAR-POP	Low Res.	14.5	100	60
	High Res.	16.2	10	62

Supplementary Table 6. Summary of low and medium resolution HadGEM3-GC31. Summary of low and medium resolution HadGEM3-GC31. The units are Sv (10^6 m^3sec^{-1}) for the AMOC, and *km* for horizontal resolution.

Model \ Description	AMOC at 26.5°N (1958-2018)	Number of Ensembles	CMIP6 Scenarios	Horizontal Resolution (Atm.)	Horizontal Resolution (Ocean)	Vertical Levels (Ocean)
HadGEM3-GC31-LL	17.7	4	SSP-585	200	100	75
HadGEM3-GC31-MM	17.5	4	SSP-585	80	25	75

References

1. Chassignet, E. P. et al. Impact of horizontal resolution on global ocean–sea ice model simulations based on the experimental protocols of the Ocean Model Intercomparison Project phase 2 (OMIP-2). *Geosci. Model Dev.* **13**, 4595–4637 (2020).
2. Hirschi, J. J.-M. et al. The Atlantic meridional overturning circulation in high-resolution models. *J. Geophys. Res.: Oceans* **125**, e2019JC015522 (2020).
3. Jones, G. S. et al. The HadGEM3-GC3.1 contribution to the CMIP6 detection and attribution model intercomparison project. *J. Adv. Model. Earth Sys.* **16**, e2023MS004135 (2024).
4. Kalnay, E. et al. The NCEP/NCAR 40-year reanalysis project. *Bull. Am. Meteor. Soc.* **77**, 437–471 (1996).
5. Kobayashi, S. et al. The JRA-55 Reanalysis: General specifications and basic characteristics. *J. Meteor. Soc. Japan* **93**, 5-48 (2015).
6. Hurrell, J. W. & Deser, C. North Atlantic climate variability: The role of the North Atlantic Oscillation. *J. Mar. Syst.* **78**, 28-41 (2009).
